# Adverse outcomes after surgeries in patients with liver cirrhosis among Korean population: A population-based study

**DOI:** 10.1371/journal.pone.0253165

**Published:** 2021-06-14

**Authors:** Hyun Ho Jo, Changwook Min, Dae-Sung Kyoung, Min-Ae Park, Sang Gyune Kim, Young Seok Kim, Young Chang, Soung Won Jeong, Jae Young Jang, Sae Hwan Lee, Hong Soo Kim, Baek Gyu Jun, Young Don Kim, Gab Jin Cheon, Jeong-Ju Yoo

**Affiliations:** 1 Department of Gastroenterology and Hepatology, Soonchunhyang University School of Medicine, Seoul, Korea; 2 Data Science Team, Hanmi Pharm. Co., Ltd., Seoul, Korea; 3 Division of Gastroenterology and Hepatology, Department of Internal Medicine, Soonchunhyang University School of Medicine, Seoul, Korea; 4 Division of Gastroenterology and Hepatology, Department of Internal Medicine, Soonchunhyang University School of Medicine, Cheonan, Korea; 5 Department of Internal Medicine, University of Ulsan College of Medicine, Gangneung Asan Hospital, Seoul, Korea; China Medical University Hospital, TAIWAN

## Abstract

**Background:**

Patients with liver cirrhosis have an increased risk of in-hospital mortality or postoperative complication after surgery. However, large-scale studies on the prognosis of these patients after surgery are lacking. The aim of the study was to investigate the adverse outcomes of patients with liver cirrhosis after surgery over five years.

**Methods and findings:**

We used the Health Insurance Review and Assessment Service-National Inpatient Samples (HIRA-NIS) between 2012 and 2016. In-hospital mortality and hospital stay were analyzed using the data. Mortality rates according to the surgical department were also analyzed. Of the 1,662,887 patients who underwent surgery, 16,174 (1.0%) patients had cirrhosis. The in-hospital mortality (8.0% vs. 1.0%) and postoperative complications such as respiratory (6.0% vs. 5.3%) or infections (2.8% vs. 2.4%) was significantly higher in patients with cirrhosis than in those without cirrhosis. In addition, the total hospitalization period and use of the intensive care unit were significantly higher in patients with liver cirrhosis. In propensity score matching analysis, liver cirrhosis increased the risk of adverse outcome significantly [adjusted OR (aOR) 1.67, 95% CI 1.56–1.79, P<0.001], especially in-hospital mortality. In liver cirrhosis group, presence of decompensation or varices showed significantly increased postoperative complication or mortality. Adverse outcomes in patients with cirrhosis was the highest in patients who underwent otorhinolaryngology surgery (aOR 1.86), followed by neurosurgery (aOR 1.72), thoracic and cardiovascular surgery (aOR 1.56), and plastic surgery (aOR 1.36).

**Conclusion:**

The adverse outcomes of patients with cirrhosis is significantly high after surgery, despite advances in cirrhosis treatment.

## Introduction

The true prevalence of liver cirrhosis in the United States is difficult to determine since well-compensated, asymptomatic patients are easily undiagnosed. However, in 2015, Mellinger et al. suggested that at least 0.27% of the American adults with private health insurance had liver cirrhosis [1]. Moreover, the incidence of liver cirrhosis is increasing due to the increased prevalence of nonalcoholic fatty liver disease [2]. In recent years, effective antiviral agents and other treatment options for liver cirrhosis have contributed to increasing the life expectancy of these patients compared to any previous era [3,4]. Therefore, compared to the past, patients with liver cirrhosis are increasingly more likely to undergo surgery. The types of surgery for patients with cirrhosis are also becoming more diverse.

An accurate evaluation of liver function is necessary prior to surgical procedures with general anesthesia since the risk of surgery is affected by liver function [5]. It is well known that perioperative morbidity, mortality and morality in ICU in patients with cirrhosis is much higher than in a control group [6–8]. Therefore, the risk-benefit assessment should be scrutinized before the decision to operate to avoid unnecessary adverse events. Indeed, the most frequent reason hepatologists are consulted by other departments is related to preoperative evaluations. To date, the Mayo score has been widely used to assess the postoperative risk of patients with liver cirrhosis [9]. However, the Mayo score was based on a formula reported in 2007, and since then, there have been very few studies on the risk of liver cirrhosis surgery [9]. Most of these studies included small numbers, single-center experiences, and showed inconsistent results. In clinical practice, we often experience poor postoperative outcomes in patients classified as low risk by Mayo scores.

Therefore, the aim of this was i) to evaluate the in-hospital mortality of patients with liver cirrhosis after surgery, and ii) identify the risk factors using a nationwide population-based study over five years.

## Materials and methods

### Data source

The Republic of Korea has a universal health coverage system with mandatory social health insurance. National health insurance in Korea covers 98% of the total population, and the number of patients claiming health insurance per year is about 46 million, reaching 90% of the resident registered population. Claims data of the Health Insurance Review and Assessment Service (HIRA) are an important source of information for healthcare service research. Claims data are generated when healthcare service providers submit a claim to HIRA for review for reimbursement. To improve the accessibility to HIRA data for healthcare service researchers, HIRA extracted 13% of the data into a national inpatient sample data (HIRA-NIS) set using a stratified randomized sampling method. The HIRA-NIS data consists of five tables, a table of general information containing demographic information such as gender and age, indicators for inpatient and outpatient services, a table of specific information on services provided, a table of diagnostic information, and a table of outpatient prescriptions.

### Study sample

**Fig 1:**
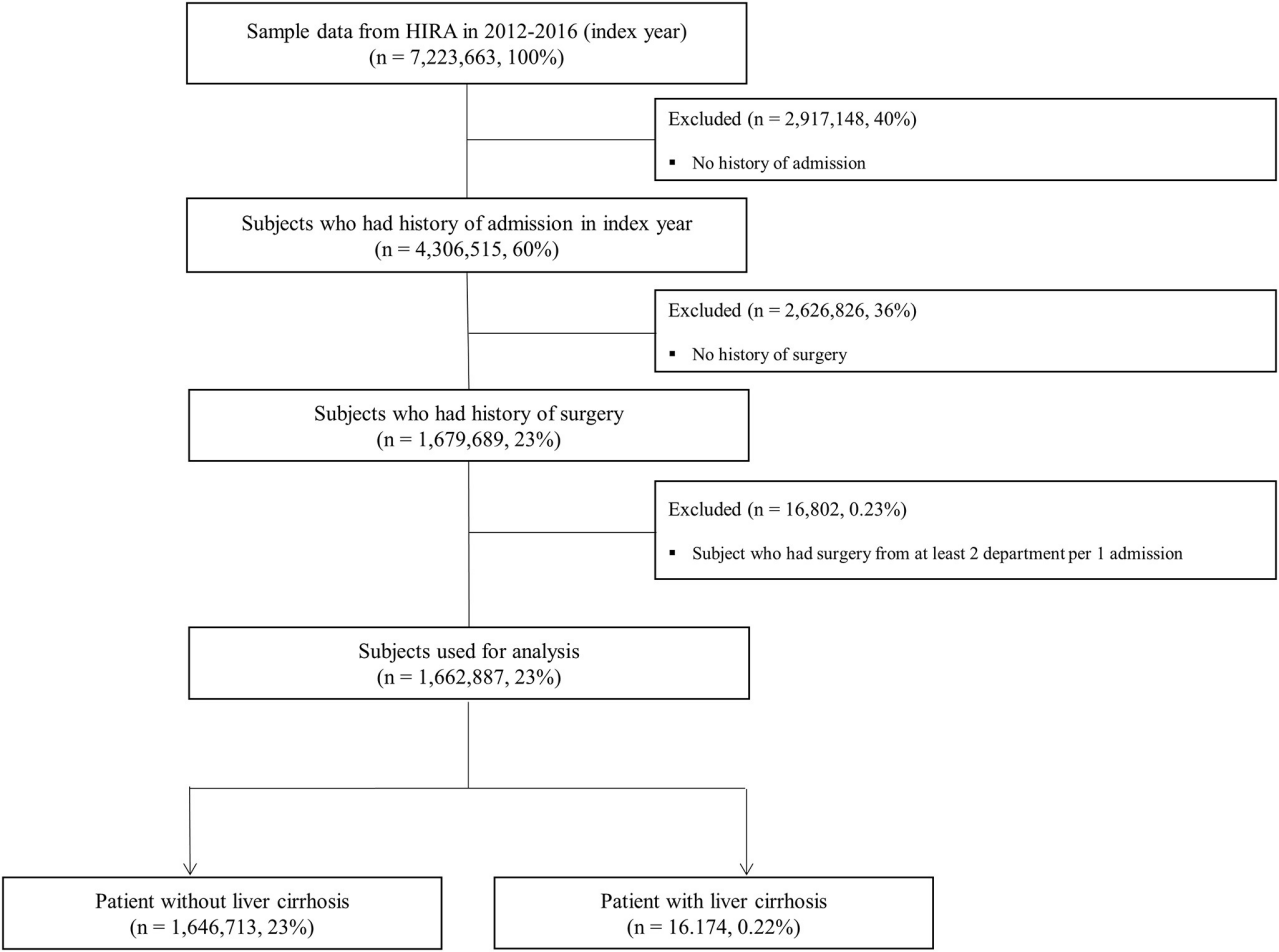
Flow chart of study sample selection.

A flow chart of the study sample selection is shown in Fig 1. First, we obtained HIRA-NIS data for five years, from 2012 to 2016 (databases accessed: 1-May-2020). Among the subjects, 16,662,887 patients were classified as having undergone surgery during that period. The patients who underwent surgery were classified into two groups according to the diagnosis of cirrhosis before surgery, i) patients with liver cirrhosis and ii) patients without liver cirrhosis. The study protocol was approved by the Institutional Review Board of Soonchunhyang University Bucheon Hospital (SCHBC 2020-04-021, date of registration 23-Apr-2020), and conformed to the ethical guidelines of the World Medical Association Declaration of Helsinki. The requirement for informed consent from individual subjects was waived due to the retrospective nature of the study.

### Definition of liver cirrhosis, severity and comorbidities

Liver cirrhosis was defined by at least one occurrence of the ICD-10 diagnosis codes K746 or K703 before surgery, regardless of the period. Decompensated cirrhosis was defined as the presence of any of the following procedures, medications, and diagnostic codes; procedure (abdominal paracentesis, endoscopic treatment of esophageal or gastric varices, sclerotherapy), medications (spironolactone, terlipressin, other systemic hemostatics, somatostatin, propranolol), diagnostic code (hepatorenal syndrome, other peritonitis, hepatic failure unspecified, oesophageal varices with bleeding in diseases classified elsewhere) [10].

We also investigated the presence of 11 comorbidities other than liver cirrhosis according to ICD-10 codes: 1) hypertension (I10, I101, I109), 2) diabetes (E10-E14), 3) any type of malignancy (all type of C code), 4) end-stage renal disease (N185-N186), 5) heart failure (I50), 6) chronic obstructive pulmonary disease (COPD) (J43-J44), 7) hyperlipidemia (E78), 8) mental disorder (F00-F99), 9) ischemic heart disease (I20-I25), 10) Parkinson’s disease (G20), and 11) Systemic Lupus Erythematosus (M32). Also, the Charlson comorbidity index was calculated [11].

### Study outcome

In the case of NIRA-NIS, it is possible to know whether the patient died during hospitalization, but the data does not provide information on whether death occurred during a follow-up period. Therefore, the primary endpoint of this study was in-hospital mortality at the time of hospitalization due to surgery. Secondary outcomes were postoperative complications. The types of complications were classified as follows; respiratory, cardiac, infections, surgical wound rupture, nervous system, bleeding or embolism [12]. The definition of postoperative complication is described in S1 Table [12].

### Statistical analysis

Frequencies and percentages are used for the descriptive statistics. Significant differences between the groups were investigated using the χ2 test for categorical variables and Student’s t-test for continuous variables. Logistic regression analysis was used to evaluate the relationships between in-hospital mortality or complications and other factors. Regression analysis was used to evaluate the relationship of medical cost and other factors. Propensity score matching (PSM) analysis was performed to minimize the probability of selection bias by pairing the liver cirrhosis (+) group and the liver cirrhosis (-) group based on propensity scores. The PSM model included clinical variables with relevance to in-hospital mortality (age, sex, medical insurance state, variable comorbidities, level of hospital, types of anesthesia, and department of surgery). We used the nearest available matching (1: 1) method for the estimated PSM. All statistical analyses were performed using R version 4.3.1 (The R Foundation for Statistical Computing, Vienna, Austria). Statistical significance was set at *P* < 0.05.

## Results

### Baseline characteristics

The characteristics of the participants classified by the presence of liver cirrhosis are described in Table 1. The flow chart of the patients analyzed in the study is presented in Fig 1. During this period, a total of 1,662,887 patients underwent surgery, of which 16,174 (0.97%) were patients with liver cirrhosis. In the group with liver cirrhosis, the average age of the patients was about 10 years older than the patients in the group without liver cirrhosis (*P*<0.001), and the proportion of men was 71%, which was higher than in the group without liver cirrhosis (*P*<0.001). In addition, the proportion of veterans or those with medical assistance, whose economic level was not high, was 12.32%, which was significantly higher than that of the group without liver cirrhosis at 4% (*P*<0.001). There was no difference in the proportion of comorbidities between the groups with and without cirrhosis. The Charlson comorbidity index also showed no difference between the two groups (0.91 vs 0.93, *P* = 0.238). As a comorbid disease, hypertension was the highest (about 17% in both groups), followed by diabetes (10.4%).

**Table 1:** Baseline characteristics of the patients.

Variable	Total (N = 1,662,887)	Liver cirrhosis (-) (N = 1,646,713)	Liver cirrhosis (+) (N = 16,174)	
Age (year)	50.06 ± 20.02	49.96 ± 20.06	60.49 ± 11.65	<0.001
Sex (n, %)				<0.001
Male	758,536 (45.62)	746,996 (45.36)	11,540 (71.35)	
Female	904,351 (54.38)	899,717 (54.64)	4,634 (28.65)	
Medical insurance state (n, %)				<0.001
Health insurance	1,593,887 (95.85)	1579706 (95.93)	14,181 (87.68)	
Veterans or medical assistance	69,000 (4.15)	67007 (4.06)	1,993 (12.32)	
Comorbidities (n, %)				
Charlson comorbidity index	0.91 ± 1.51	0.91 ± 1.51	0.93 ± 1.54	0.238
Hypertension	287,030 (17.26)	284163 (17.25)	2,867 (17.73)	0.118
Diabetes	174,572 (10.50)	172884 (10.49)	1,688 (10.44)	0.807
Malignancy	59,143 (3.56)	58513 (3.55)	630 (3.90)	0.021
End stage renal disease	2,729 (0.16)	2696 (0.16)	33 (0.20)	0.244
Chronic obstructive pulmonary disease	21,743 (1.31)	21526 (1.30)	217 (1.34)	0.727
Heart failure	20,931 (1.26)	20723 (1.25)	208 (1.29)	0.781
Hyperlipidemia	309540(18.61)	306549(18.62)	2991(18.49)	0.688
Mental disorder	253143(15.22)	250661(15.22)	2482(15.35)	0.671
Ischemic heart disease	69272(4.17)	68609(4.17)	663(4.1)	0.684
Parkinson’s disease	5323(0.32)	5266(0.32)	57(0.35)	0.508
Systemic Lupus Erythematosus	2130(0.13)	2111(0.13)	19(0.12)	0.788
Level of hospital (n, %)				<0.001
Primary clinic	1544865(93.3)	1530162(93.32)	14703(91.34)	
Secondary hospital	98634(5.96)	97398(5.94)	1236(7.68)	
Tertiary hospital	12248(0.74)	12090(0.74)	158(0.98)	
Missing	7140			
Types of anesthesia (n, %)				<0.001
Non-general anesthesia	1127999(67.83)	1119093(67.96)	8906(55.06)	
General anesthesia	534888(32.17)	527620(32.04)	7268(44.94)	
Department of surgery (n, %)				
Orthopedic surgery	397,700 (23.92)	394803 (23.98)	2,897 (17.91)	<0.001
Ophthalmology	246,197 (14.81)	244118 (14.82)	2,079 (12.85)	<0.001
Plastic surgery	150,611 (9.06)	146113 (8.87)	4,498 (27.81)	<0.001
Dental surgery	6,154 (0.37)	6090 (0.36)	64 (0.40)	0.635
Obstetrics and gynecology	191,168 (11.50)	190902 (11.59)	266 (1.64)	<0.001
Otorhinolaryngology	152,180 (9.15)	151256 (9.18)	924 (5.71)	<0.001
Cardiothoracic surgery	51,794 (3.11)	50947 (3.09)	847 (5.24)	<0.001
Neurosurgery	148,244 (8.91)	146784 (8.91)	1,460 (9.03)	0.625
General surgery	391,777 (23.56)	386490 (23.47)	5,287 (32.69)	<0.001
Urology	38,858 (2.34)	38604 (2.34)	254 (1.57)	<0.001

NOTE. Data are presented as mean ± standard deviation for continuous variables and n (%) for categorical variables.

Abbreviations: N, number.

When classified at the hospital level, patients with cirrhosis had a higher rate of undergoing surgery in secondary or tertiary hospitals compared to the control group (8.66% vs. 6.68%, *P* < 0.001). In addition, liver cirrhosis patients had a significantly higher rate of general anesthesia than the control group (44.9% vs. 32.0%, *P* < 0.001).

There were differences in the type of surgery between the group with cirrhosis and the group without cirrhosis. In the group without cirrhosis, orthopedic surgery was the highest, followed by general surgery. In the group with cirrhosis of the liver, general surgery was the highest, followed by plastic surgery. Among the types of general surgery, cholecystectomy was common, and during plastic surgery, debridement and escharectomy due to pressure ulcers were common. The names of the top three surgeries by year, liver cirrhosis status, and surgical procedures are described in the supplementary material (S2 Table).

When cirrhotic patients were classified into compensated and decompensated according to liver function, the number of decompensated patients was 1102, corresponding to 6.8%.

### Postoperative outcomes according to the presence of liver cirrhosis

The characteristics of the participants classified by the presence of liver cirrhosis are described in Table 1. The clinical outcomes after surgery are listed in Table 2. The average length of hospital stay was significantly longer in the liver cirrhosis (+) group (22.44 days vs 10.18 days, *P* < 0.001). Also, the medical cost of hospitalization was higher in the liver cirrhosis (+) group. In the liver cirrhosis group, the rate of admission to the intensive care unit after surgery was significantly higher than in the group without cirrhosis (24.3% vs. 4.8%, *P* < 0.001).

**Table 2:** Clinical outcomes of the patients.

Variables	Total (N = 1,662,887)	Liver cirrhosis (-) (N = 1,646,713)	Liver cirrhosis (+) (N = 16,174)	
Average length of hospital stay (days)	10.31± 26.68	10.18 ± 26.42	22.44 ± 43.11	<0.001
Average cost of hospitalization (won)	4,041,522.51 ± 16,545,326.08	3,928,082 ± 15,618,768	14,674,804± 54,342,232	<0.001
Intensive care unit admission rate (n, %)	83,930 (5.05)	80000 (4.86)	3930 (24.30)	<0.001
Post-operative complication (n, %)				
Respiratory	89683 (5.39)	88713 (5.39)	970 (6)	0.001
Cardiac	43512 (2.62)	43058 (2.61)	454 (2.81)	0.134
Infections	40573 (2.44)	40107 (2.44)	466 (2.88)	<0.001
Surgical wound rupture	176 (0.01)	175 (0.01)	1 (0.01)	1.000
Nervous system	17482 (1.05)	17325 (1.05)	157 (0.97)	0.331
Bleeding	449 (0.03)	442 (0.03)	7 (0.04)	0.219
Embolism	1694 (0.1)	1681 (0.1)	13 (0.08)	0.461
In-hospital mortality (n, %)	18,814 (1.13)	17,520 (1.06)	1,294 (8.00)	<0.001

NOTE. Data are presented as mean ± standard deviation for continuous variables and n (%) for categorical variables.

Abbreviations: N, number.

When examining the incidence of postoperative complications, the incidence of complications of the respiratory system (6.0% vs. 5.4%, *P* = 0.001) and postoperative infection (2.9% vs. 2.4%, *P* < 0.001) was higher in the liver cirrhosis group than in the control group. However, the incidence of cardiac complication, surgical wound rupture, nervous system, bleeding, and embolism after surgery showed no difference between the two groups.

Lastly, postoperative in-hospital mortality was significantly higher in the cirrhosis group (8.0%) than in the group without cirrhosis (1.0%).

### Propensity score matching analysis

The group with cirrhosis had a poor postoperative clinical prognosis and higher in-hospital mortality than the group without cirrhosis. However, since the patients in the cirrhosis group were more than 10 years older than those in the non-cirrhosis group, it is difficult to determine whether these findings were due to age or liver cirrhosis. Therefore, PSM was performed to adjust for factors in addition to liver cirrhosis that may have affected the clinical outcomes or in-hospital mortality. Table 3 shows the PSM results matched for age, sex, medical insurance state, variable comorbidities, level of hospital, types of anesthesia, and department of surgery. In the matched set, there was no significant difference in the underlying characteristics between the two groups.

**Table 3:** Baseline characteristics and clinical outcomes of the matched set.

Variable	Total	Liver cirrhosis (-)	Liver cirrhosis (+)	p-value
	(N = 32,194)	(N = 16,097)	(N = 16,097)	
Age (year)	60.5 ± 11.78	60.52 ± 11.91	60.48 ± 11.65	0.808
Sex (n, %)				0.844
Male	22954(71.3)	11469(71.25)	11485(71.35)	
Female	9240(28.7)	4628(28.75)	4612(28.65)	
Medical insurance state (n, %)				0.905
Health insurance	28234(87.7)	14121(87.72)	14113(87.67)	
Veterans or medical assistance	3960(12.3)	1976(12.28)	1984(12.33)	
Comorbidities (n, %)				
Charlson comorbidity index	0.92 ± 1.53	0.92 ± 1.52	0.93 ± 1.54	0.644
Hypertension	5682(17.65)	2830(17.58)	2852(17.72)	0.759
Diabetes	3370(10.47)	1692(10.51)	1678(10.42)	0.813
Malignancy	1232(3.83)	604(3.75)	628(3.9)	0.504
End stage renal disease	65(0.2)	33(0.21)	32(0.2)	1.000
Chronic obstructive pulmonary disease	424(1.32)	210(1.3)	214(1.33)	0.883
Heart failure	394(1.22)	188(1.17)	206(1.28)	0.389
Hyperlipidemia	5953(18.49)	2976(18.49)	2977(18.49)	1.000
Mental disorder	4932(15.32)	2462(15.29)	2470(15.34)	0.914
Ischemic heart disease	1312(4.08)	651(4.04)	661(4.11)	0.800
Parkinson’s disease	97(0.3)	41(0.25)	56(0.35)	0.155
Systemic Lupus Erythematosus	34(0.11)	15(0.09)	19(0.12)	0.607
Level of hospital				
Primary clinic	29423(91.39)	14720(91.45)	14703(91.34)	0.943
Secondary hospital	2458(7.63)	1222(7.59)	1236(7.68)	
Tertiary hospital	313(0.97)	155(0.96)	158(0.98)	
Types of anesthesia				
Non-general anesthesia	17677(54.91)	8824(54.82)	8853(55)	0.754
General anesthesia	14517(45.09)	7273(45.18)	7244(45)	
Department of surgery (n, %)				
Orthopedic surgery	5725(17.78)	2840(17.64)	2885(17.92)	0.521
Ophthalmology	4134(12.84)	2065(12.83)	2069(12.85)	0.960
Plastic surgery	8912(27.68)	4443(27.6)	4469(27.76)	0.756
Dental surgery	124(0.39)	60(0.37)	64(0.4)	0.787
Obstetrics and gynecology	528(1.64)	263(1.63)	265(1.65)	0.965
Otorhinolaryngology	1812(5.63)	892(5.54)	920(5.72)	0.514
Cardiothoracic surgery	1688(5.24)	843(5.24)	845(5.25)	0.980
Neurosurgery	2864(8.9)	1414(8.78)	1450(9.01)	0.493
General surgery	10518(32.67)	5249(32.61)	5269(32.73)	0.821
Urology	518(1.61)	264(1.64)	254(1.58)	0.690

*Matching variable: Age, Sex, Medical insurance state, Hypertension, Diabetes, Malignancy, End stage renal disease, Chronic obstructive pulmonary disease, Heart failure, Hyperlipidemia, Mental disorder, Ischemic heart disease, Parkinson’s disease, Systemic Lupus Erythematosus, Level of hospital, Types of anesthesia, Department of surgery.

### Impact of liver cirrhosis on the adverse outcomes (postoperative complications or mortality) and consumption of medical resources

Adverse outcomes were defined in-hospital mortality or postoperative complications. Multivariate analysis of the factors affecting adverse outcome was performed for the entire patient group and matched set (Table 4). After matching, liver cirrhosis significantly increased the risk of postoperative complications and mortality compared to the group without cirrhosis [adjusted odds ratio (OR) 1.67, 95% confidence interval (CI) 1.56–1.79, *P*<0.0001].

**Table 4:** Multivariate logistic regression predicting adverse outcome (mortality or post-operative complication) of all patients.

Variables	Unmatched set (N = 1,662,887)				Matched set (N = 32,194)			p-value
	Univariate		Multivariate		Univariate		Multivariate	
	OR (95% CI)	p-value	OR (95% CI)	p-value	OR (95% CI)	p-value	OR (95% CI)	
Age (year)	1.01(1.01–1.01)	<.0001	1.01(1.01–1.01)	<.0001	1.01(1–1.02)	0.164		
Sex								
Female	1 (Ref)							
Male	1.06(1.05–1.07)	<.0001	1.07(1.06–1.09)	<.0001	1.04(0.71–1.53)	0.843		
Medical insurance state								
Health insurance	1 (Ref)							
Veterans or medical assistance	1.28(1.25–1.31)	<.0001	1.21(1.18–1.24)	<.0001	0.84(0.6–1.19)	0.3334		
Comorbidities (n, %)								
Charlson comorbidity index	1.51(1.5–1.51)	<.0001	1.48(1.47–1.49)	<.0001	1.44(1.37–1.5)	<.0001	1.47(1.4–1.55)	<.0001
Liver cirrhosis	1.77(1.7–1.84)	<.0001	1.68(1.61–1.76)	<.0001	1.61(1.51–1.72)	<.0001	1.67(1.56–1.79)	<.0001
Hypertension	3.44(3.41–3.48)	<.0001	0.75(0.74–0.76)	<.0001	2.4(1.71–3.38)	<.0001	1.19(0.78–1.82)	0.4276
Diabetes	3.12(3.08–3.16)	<.0001	1.78(1.75–1.81)	<.0001	1.39(1.01–1.9)	0.0407	0.46(0.29–0.73)	0.0008
Malignancy	2.27(2.22–2.32)	<.0001	3.14(3.05–3.23)	<.0001	1.12(0.77–1.63)	0.5639		
End stage renal disease	5.92(5.49–6.39)	<.0001	2.02(1.84–2.23)	<.0001	1(0.46–2.16)	1		
Chronic obstructive pulmonary disease	7(6.81–7.19)	<.0001	2.27(2.2–2.35)	<.0001	3.24(1.88–5.57)	<.0001	1.75(0.89–3.43)	0.1057
Heart failure	14.99(14.57–15.43)	<.0001	0.38(0.37–0.39)	<.0001	4.56(2.66–7.84)	<.0001	1.88(1–3.52)	0.0506
Hyperlipidemia	3.11(3.08–3.14)	<.0001	1.09(1.07–1.11)	<.0001	1.86(1.34–2.57)	0.0002	1.16(0.74–1.83)	0.5175
Mental disorder	2.72(2.69–2.75)	<.0001	1.23(1.21–1.25)	<.0001	1.49(1.09–2.04)	0.0125	0.77(0.51–1.17)	0.2281
Ischemic heart disease	11.18(11–11.36)	<.0001	4.57(4.48–4.66)	<.0001	3.43(2.29–5.16)	<.0001	2.16(1.31–3.58)	0.0027
Parkinson’s disease	4.42(4.18–4.68)	<.0001	1.26(1.18–1.35)	<.0001	2.08(1.05–4.15)	0.0366	2.05(0.81–5.18)	0.1296
Systemic Lupus Erythematosus	2.18(1.97–2.42)	<.0001	0.8(0.71–0.9)	0.0002	0.8(0.22–2.98)	0.7394		
Level of hospital								
Primary hospital	1 (Ref)							
Secondary hospital	1.05(1.03–1.07)	<.0001	1.04(1.02–1.06)	0.0004	0.63(0.42–0.93)	0.0202	0.93(0.56–1.57)	0.7922
Tertiary Hospital	1.09(1.04–1.15)	0.0013	1.06(1–1.13)	0.0512	0.58(0.26–1.29)	0.1798	0.75(0.29–1.98)	0.5642
Types of anesthesia								
Non-General anesthesia	1 (Ref)							
General anesthesia	1.11(1.1–1.13)	<.0001	1.09(1.08–1.11)	<.0001	0.94(0.67–1.32)	0.7297		
Department of surgery (n, %)								
Orthopedic surgery	0.98(0.97–0.99)	<.0001	1.04(1.02–1.05)	<.0001	1.17(0.79–1.72)	0.4332		
Ophthalmology	0.97(0.96–0.99)	0.0002	0.97(0.96–0.99)	0.0034	0.82(0.51–1.31)	0.4069		
Plastic surgery	1.23(1.21–1.25)	<.0001	1.23(1.21–1.26)	<.0001	1.15(0.8–1.65)	0.4618		
Dental surgery	1.11(1.03–1.2)	0.0079	1.13(1.04–1.23)	0.0031	0.5(0.2–1.24)	0.1343		
Obstetrics and gynecology	0.88(0.86–0.89)	<.0001	1.11(1.09–1.14)	<.0001	0.77(0.34–1.75)	0.5328		
Otorhinolaryngology	1.13(1.11–1.15)	<.0001	1.29(1.27–1.32)	<.0001	1.35(0.88–2.07)	0.165		
Cardiothoracic surgery	1.4(1.37–1.43)	<.0001	1.46(1.42–1.5)	<.0001	0.86(0.53–1.39)	0.5417		
Neurosurgery	1.2(1.18–1.22)	<.0001	1.19(1.17–1.22)	<.0001	0.95(0.61–1.49)	0.8186		
General surgery	1(0.99–1.01)	0.5231			1.18(0.84–1.64)	0.3478		
Urology	0.97(0.94–1)	0.0626			0.56(0.26–1.2)	0.1361		

*Matching variable: Age, Sex, Medical insurance state, Hypertension, Diabetes, Malignancy, End stage renal disease, Chronic obstructive pulmonary disease, Heart failure, Hyperlipidemia, Mental disorder, Ischemic heart disease, Parkinson’s disease, Systemic Lupus Erythematosus, Level of hospital, Types of anesthesia, Department of surgery.

When the adverse outcomes were divided into in-hospital mortality and postoperative complication, especially in the liver cirrhosis group, in-hospital mortality was significantly increased by more than 3 times (adjusted OR 3.97, 95% CI 3.50–4.49, *P* < 0.001) (S3 Table). On the other hand, the incidence of postoperative complication was not significant in the liver cirrhosis group than control group times (adjusted OR 1.08, 95% CI 1–1.16, *P* = 0.052) (S4 Table).

Finally, the cost of hospitalization was significantly higher in cirrhosis compared to the control group (beta coefficient 7839090, *P* < 0.001) (S5 Table).

### Severity of liver cirrhosis on the adverse outcomes (postoperative complications or mortality)

Next, we investigated the impact of severity of liver disease on the mortality or postoperative complications in patients with liver cirrhosis. For the evaluation of liver disease severity, presence of decompensation, ascites, varices, chronic hepatitis B, chronic hepatitis C were used. In multivariate analysis, presence of decompensation (adjusted OR 1.26, 95% CI 1.06–1.50, P = 0.009) or presence of varices (adjusted OR 1.41, 95% CI 1.19–1.66, P<0.0001) were related with mortality or post-operative complication in cirrhotic patients (Table 5).

**Table 5:** Multivariate logistic regression predicting adverse outcome (mortality or post-operative complication) in patients with liver cirrhosis.

Patients with liver cirrhosis (N = 16,174)				
Variables	Univariate		Multivariate	
	OR (95% CI)	p-value	OR (95% CI)	p-value
**Age (year)**	1.01(1.01–1.01)	<.0001	1.01(1.01–1.02)	<.0001
**Sex**				
Female	1 (Ref)		1 (Ref)	
Male	1.12(1.02–1.22)	0.0144	1.16(1.05–1.27)	0.0032
**Medical insurance state**				
Health insurance	1 (Ref)			
Veterans or medical assistance	1.09(0.97–1.23)	0.1419		
**Comorbidities (n, %)**	1.34(1.31–1.38)	<.0001	1.36(1.3–1.41)	<.0001
Charlson comorbidity index				
Hypertension	2.34(2.13–2.56)	<.0001	1.13(1–1.28)	0.0556
Diabetes	2.19(1.95–2.44)	<.0001	0.63(0.53–0.74)	<.0001
Malignancy	1.54(1.28–1.85)	<.0001	0.34(0.27–0.44)	<.0001
End stage renal disease	2.55(1.25–5.18)	0.01	0.42(0.18–0.99)	0.046
Chronic obstructive pulmonary disease	4.45(3.4–5.82)	<.0001	1.88(1.37–2.57)	<.0001
Heart failure	10.45(7.76–14.07)	<.0001	2.41(1.71–3.39)	<.0001
Hyperlipidemia	2.19(2–2.4)	<.0001	1.07(0.94–1.21)	0.3074
Mental disorder	1.87(1.69–2.06)	<.0001	1.09(0.97–1.22)	0.1625
Ischemic heart disease	7.6(6.47–8.93)	<.0001	3.94(3.26–4.75)	<.0001
Parkinson’s disease	4.03(2.39–6.78)	<.0001	1.33(0.72–2.45)	0.3585
Systemic Lupus Erythematosus	2.6(1.02–6.6)	0.0452	1.4(0.5–3.89)	0.5213
**Level of hospital**				
Primary hospital (= Clinic)	1 (Ref)			
Secondary hospital (= Hospital, General hospital)	0.93(0.8–1.09)	0.3815		
Tertiary Hospital	1.31(0.9–1.9)	0.1583		
**Types of anesthesia**				
Non-General anesthesia				
General anesthesia	0.98(0.9–1.06)	0.6099		
**Severity of liver cirrhosis**				
Decompensated liver cirrhosis	1.69(1.47–1.95)	<.0001	1.26(1.06–1.5)	0.009
Chronic hepatitis B	1.16(0.84–1.60)	0.3797		
Chronic hepatitis C	2.06(1.34–5.03)	0.0046	1.3(0.6–2.83)	0.5087
Ascites	1.52(1.15–1.99)	0.0028	0.95(0.68–1.33)	0.7771
Varices	1.42(1.22–1.66)	<.0001	1.41(1.19–1.66)	<.0001
Hepatic encephalopathy	1.37(0.45–4.20)	0.5842		
**Department of surgery (n, %)**				
Orthopedic surgery	0.99(0.89–1.09)	0.7785		
Ophthalmology	0.76(0.67–0.86)	<.0001	0.85(0.73–0.98)	0.0267
Plastic surgery	1.28(1.18–1.4)	<.0001	1.36(1.23–1.51)	<.0001
Dental surgery	0.64(0.3–1.33)	0.2292		
Obstetrics and gynecology	0.74(0.52–1.04)	0.0839		
Otorhinolaryngology	1.67(1.43–1.94)	<.0001	1.86(1.58–2.2)	<.0001
Cardiothoracic surgery	1.32(1.12–1.56)	0.0009	1.56(1.31–1.86)	<.0001
Neurosurgery	1.57(1.38–1.78)	<.0001	1.72(1.49–1.99)	<.0001
General surgery	0.83(0.76–0.91)	<.0001	1.01(0.91–1.12)	0.8661
Urology	0.71(0.49–1.01)	0.0581		

Abbreviations: N, number; OR, odds ratio; CI, confidence interval.

In addition to these factors, older age (adjusted OR 1.01), male gender (adjusted OR 1.16), and multiple comorbidities such as diabetes, chronic obstructive pulmonary disease, heart disease were risk factors for adverse outcomes after surgery. In addition, among the types of surgery, otorhinolaryngology (adjusted OR 1.86), neurosurgery (adjusted OR 1.72), cardiothoracic surgery (adjusted OR 1.56), and plastic surgery (adjusted OR 1.36) showed higher postoperative mortality than other types of surgery (Table 5).

When the adverse outcomes were divided into postoperative complication (S6 Table) and in-hospital mortality (S7 Table), the presence of decompensation increased both morality and postoperative complication, but the presence of varices increased mortality, but was not related to postoperative complication.

## Discussion

In this nationwide population-based study, we found that the in-hospital mortality after surgery in patients with cirrhosis increased by about five times compared to the patients without cirrhosis, and additionally found that the risk varied according to age, sex, and the type of surgery.

Previous studies showed that the burden of liver cirrhosis is growing regardless of the region [13]. In particular, with the recent use of effective therapeutic agents for hepatitis B and hepatitis C, the likelihood of liver cirrhosis patients surviving to old age is higher than previously. Therefore, patients with cirrhosis have a greater chance of undergoing a variety of surgeries, just like patients without cirrhosis. In fact, the most frequent reason hepatologists at tertiary hospitals are consulted by other departments is related to the risk of surgery in patients with cirrhosis. However, the interest of hepatologists on the topic of surgical risk in patients with cirrhosis is relatively low, so the number of related studies is insufficient compared to other topics.

The results of published studies indicated that the postoperative mortality rate for general patients without cirrhosis is 1.1%, whereas the mortality rate for patients with cirrhosis is 8.3–25% [14,15]. The factors influencing the risk of surgery in patients with cirrhosis are liver function and the type of surgery [16,17]. In particular, it is known that the Child-Pugh and MELD scores are related to postoperative patient prognosis [18–20]. Since the paper published by Swee *et al*. in 2007, the Mayo score is now widely used in clinical practice [9]. However, the Mayo score is a scoring system for digestive, orthopedic, and cardiovascular surgery only, and is difficult to apply to other types of surgery. In addition, a study reported that this scoring system model tended to overestimate mortality, especially one year after surgery [21]. This is probably because of improvements in the overall care of critically ill patients since the mid-2000s [19]. Recent studies are difficult to generalize because of the relatively small numbers, single-center studies, and analyses of limited types of surgery. Our study was the first to identify the risk of surgery in patients with cirrhosis using large-scale data from a nationwide population.

In our study, the in-hospital mortality of the patients with cirrhosis was 8%. Compared to the general population, it was more than seven times higher, and even after age-sex correction, it was more than five times higher. In addition, the rate of admission to the intensive care unit, the duration of hospitalization, and hospitalization costs were all significantly higher. Considering that previous studies reported that the in-hospital mortality rate of liver cirrhosis patients was 8.3–25%, the postoperative outcomes seem to have improved compared to the previous reports. This can be explained by two factors. The first is that the recent intraoperative and postoperative treatment for patients with cirrhosis of the liver has advanced, and the second is that most of the patients who have already undergone surgery are likely to have compensated cirrhosis and thus, relatively good liver function.

In our study, the risk factors for postoperative in-hospital mortality in patients with cirrhosis were older age, male gender, low SES, and certain types of surgery. Old age is a risk factor already identified by other studies, and the other risk factors were newly identified in this study [9]. Low SES status can result in malnutrition. Indeed, malnutrition or hypoalbuminemia has already been reported as risk factors in previous studies [22].

Regarding the type of surgery, the risk of otorhinolaryngology, neurosurgery, and cardiothoracic surgery was high in our study. First, in relation to otolaryngology surgery, several small-scale head and neck cancer cohort results have been reported. In the previous study, the mortality rate of the group with liver disease was reported to be about 6 times higher than that of the control group, which is considerably higher than that of our study 3.8 times [23]. In our study, the proportion of tracheotomies was the highest among the otolaryngology surgery. Second, in the case of neurosurgery, the results of brain surgery in patients with cirrhosis have been reported. Overall, the risk of brain surgery in cirrhotic patients was very high, and mortality was reported at 24% and morbidity at 52.1% [24]. Even in child A, the complication rate was 38.7%, and the risk increased as the chilld-pugh score increased [24]. In our study, the mortality increased by more than about twice, and this is the same conclusion as the previous study. Third, cardiovascular surgery, which was the highest risk in our study, was reported as the same high risk in other studies. Generally, thoracic surgery was classified as a high-risk surgery, attributed to the hemodynamic alterations in patients with cirrhosis [25–27]. Recently, cardiovascular disease has increased in patients with liver cirrhosis caused by fatty liver. But studies are limited about intraoperative and postoperative issues. Overall, mortality and morbidity were high, but varying from study to study, and reported up to 4–70% [5].

On the other hand, in the case of general surgery or orthopedics, there was no significant difference in in-hospital mortality. The general surgery classified in this study include various types of surgery, and the risk is reported differently depending on the type of surgery. It has been reported that there is no difference in mortality in cholecystectomy or hernia operation compared to the control group, and it is performed commonly in clinical practice [5,28]. Meanwhile, other abdominal surgery including pancreatic surgery was classified as high risk [9,29,30]. In our study, the risk of in-hospital mortality in the patients undergoing general surgery, who mainly received intraperitoneal surgery, was rather low compared to patients undergoing surgery in other surgical departments. In this study, it is likely that the rate of general surgery belonging to the low-risk group was high.

Meanwhile, the high risk of plastic surgery was newly found in our study. Among plastic surgery, debridement, and escharectomy due to pressure ulcers were most frequently performed, and the risk of mortality after surgery was probably increased by patient factors rather than the type of surgery.

The items used in the Mayo score are age, American Society of Anesthesiologists score, and scoring system related to liver function evaluation. The results of our study indicate that sex, socioeconomic status, and the type of surgery should be reflected in the surgical risk assessment. If these items are added to the existing Mayo score, it will be possible to more accurately predict the surgical risk of patients with cirrhosis.

Our study had several limitations. First of all, there is an inevitable selection bias arising from the retrospective design. Since this study defined liver cirrhosis based on the diagnosis code, it is possible that cirrhosis that was not diagnosed before surgery was not included. In addition, patients with advanced liver cirrhosis are often unable to perform surgery, and sicker patients might excluded from the beginning. Therefore, only patients with relatively well-preserved liver function are selectively included, and there is a possibility of risk underestimation. And out-patient surgery was not included in this study.

Second, there is no information on the Child-Pugh or MELD scores. It was difficult to calculate accurate liver function because we could not get the information of blood test, ascites or hepatic encephalopathy. Instead, we tried to overcome this problem by using the definition of decompensated cirrhosis, which has been commonly verified in previous studies [10]. In this study, mortality was reported to be significantly higher in the decompensation group, and if MELD or child-pugh data can be obtained similar results are expected.

Third, since we classified patients into departments that performed surgery rather than the type of surgery, it was difficult to find out the risk for each type of surgery. In addition, in this study, it was difficult to distinguish from emergency surgery and elective surgery, so the effect of emergency surgery, which is known as a common risk factor in previous studies, on the mortality rate was not calculated [31–33]. Finally, there are various risk factors that influence the mortality rate after surgery other than liver cirrhosis. This study attempted to overcome this through propensity score matching, but it is possible that factors other than matching items influenced mortality. In addition, a significant correlation between liver cirrhosis and postoperative mortality could be proven, but it was difficult to see the cause-effect relation between these two factors. Overall, it may be difficult to generalize our study results to all patients with cirrhosis due to the limitations mentioned above.

In conclusion, the in-hospital mortality of patients with cirrhosis is significantly high, despite the advances in cirrhosis treatment. For precise surgical risk assessment of these patients, not only liver function, but also age, sex, and the type of surgery should be considered. In the future, an accurate formula for predicting postoperative mortality in patients with cirrhosis should be developed.

## Supporting information

S1 Table Definition of post-operative complication. (DOCX)

S2 Table Top 3 surgical indications by year, department, and liver cirrhosis. (DOCX)

S3 Table Multivariate logistic regression predicting in-hospital mortality of all patients. (DOCX)

S4 Table Multivariate logistic regression predicting post-operative complication of all patients. (DOCX)

S5 Table Multivariate linear regression analysis predicting medical cost of all patients. (DOCX)

S6 Table Multivariate logistic regression predicting post-operative complication in patients with liver cirrhosis. (DOCX)

S7 Table Multivariate logistic regression predicting in-hospital mortality in patients with liver cirrhosis. (DOCX)

## Abbreviations

HIRA: Health Insurance Review and Assessment Service
NIS: national inpatient sample data
OR: odds ratio
PSM: propensity score matching
SES: socioeconomic status
