# Supplementary material for: Adverse outcomes after surgeries in patients with liver cirrhosis among Korean population: A population-based study
Source: PLoS One. 2021 Jun 14;16(6):e0253165. doi: 10.1371/journal.pone.0253165 (PMC8202950; doi:10.1371/journal.pone.0253165)
Supplement: S1 Table — (DOCX) [file pone.0253165.s001.docx]

**Supplementary Table 1. Definition of post-operative complication**

| **Respiratory** | Pneumonia | J15, J18 |
| --- | --- | --- |
|  | Respiratory other (asthma, pleural effusion, pneumothorax, respiratory failure, pulmonary oedema, phlebitis and thrombophlebitis) | J45, J80-84, J90, J91, J93, J96, R06, R09 |
| **Cardiac** | Cardiac arrhythmia | I44, I48, I49 |
|  | Congestive heart failure | I50, I51 |
|  | Cardiac other (angina pectoris, cardiac arrest, myocardial infarction, acute ischemic heart disease, cardiovascular shock) | I20-24, I46, R96, T81.1 |
| **Infections** | Sepsis | A40, A41, R65 |
|  | Surgical site | T81.4-6, T82.7, T85.7, T88.0 |
|  | Urinary tract | N30, N39 |
|  | Infections other (other bacterial intestinal infections, E-coli, Clostridium difficile, meningitis, pericarditis, nosocomial infection) | A04, G00, I31, O86, Y95 |
| **Surgical wound rupture** | Surgical wound rupture | T81.3 |
| **Nervous system** | Delirium, somnolence, other | F05, R40, R29 |
|  | Cerebral infarction | I63 |
| **Bleeding** | Bleeding | J94, T80.3-4, T81.0, T82.8 |
| **Embolism** | Arterial-, venous-, lung- and air embolies | I26, I80, T80.0, T81.7 |
