# Supplementary material for: Adverse outcomes after surgeries in patients with liver cirrhosis among Korean population: A population-based study
Source: PLoS One. 2021 Jun 14;16(6):e0253165. doi: 10.1371/journal.pone.0253165 (PMC8202950; doi:10.1371/journal.pone.0253165)
Supplement: S2 Table — (DOCX) [file pone.0253165.s002.docx]

**Supplementary Table 2. Top 3 surgical indications by year, department, and liver cirrhosis**

**(A) Top 3 surgical indications in all patients**

| **Year** | **Name of surgery** |
| --- | --- |
| 2012 | Intraocular Lens Implantation-Primary |
| 2012 | Surgery for Cataract Or Lens-Phacoemulsification |
| 2012 | Operation of Hemorrhoids-Hemorrhoidectomy |
| 2013 | Intraocular Lens Implantation-Primary |
| 2013 | Surgery for Cataract Or Lens-Phacoemulsification |
| 2013 | Operation of Hemorrhoids-Hemorrhoidectomy |
| 2014 | Intraocular Lens Implantation-Primary |
| 2014 | Surgery for Cataract Or Lens-Phacoemulsification |
| 2014 | Operation of Hemorrhoids-Hemorrhoidectomy |
| 2015 | Intraocular Lens Implantation-Primary |
| 2015 | Surgery for Cataract Or Lens-Phacoemulsification |
| 2015 | Escharectomy[9% Under]-Hand, Foot, Finger or Toe |
| 2016 | Intraocular Lens Implantation-Primary |
| 2016 | Surgery for Cataract Or Lens-Phacoemulsification |
| 2016 | Escharectomy[9% Under]-Hand, Foot, Finger or Toe |

**(B) Top 3 surgical indications according to the department of surgery**

| **Year** | **Department of surgery** | **Name of surgery** |
| --- | --- | --- |
| 2012 | Orthopedic surgery | Menisectomy(Medial or Lateral) |
| 2012 | Orthopedic surgery | Reconstruction of Tendon And Ligament, Simple |
| 2012 | Orthopedic surgery | Replacement Arthroplasty-Total[Knee] |
| 2012 | Ophthalmology | Intraocular Lens Implantation-Primary |
| 2012 | Ophthalmology | Surgery for Cataract Or Lens-Phacoemulsification |
| 2012 | Ophthalmology | Surgery for Cataract Or Lens-Extracapsular Or Intracapsular Extraction |
| 2012 | Plastic surgery | Trigger Finger |
| 2012 | Plastic surgery | Skin Flap-Local-Others |
| 2012 | Plastic surgery | Escharectomy[9% Under]-Hand, Foot, Finger or Toe |
| 2012 | Dental surgery | Open Reduction of Mandibular Fracture(Symphysis, Body, Angle of Mandible) |
| 2012 | Dental surgery | Operation of Fractured Upper Jaw(Open Reduction, Le Fort Ⅰ) |
| 2012 | Dental surgery | Removal of The Fixation Mini Plate |
| 2012 | Obstetrics and gynecology | Cesarean Section Delivery-First Fetus-Initial-Primiparous |
| 2012 | Obstetrics and gynecology | Cesarean Section Delivery-First Fetus-Repeat |
| 2012 | Obstetrics and gynecology | Extirpation of Adnexal Tumor-Benign |
| 2012 | Otorhinolaryngology | Submucosal Resection or Septoplasty(Bone) |
| 2012 | Otorhinolaryngology | Tonsillectomy |
| 2012 | Otorhinolaryngology | Submucosal Inferior Turbinectomy |
| 2012 | Cardiothoracic surgery | Extracorporeal Circulation By Heart-Lung Machine |
| 2012 | Cardiothoracic surgery | Wedge Resection of Lung, Single |
| 2012 | Cardiothoracic surgery | Extensive Resection of Varicose Vein-Segmental Stripping of Saphenous Vein,Stab Abulsion of Varices |
| 2012 | Neurosurgery | Diskectomy(Invasive)-Lumbar Spine |
| 2012 | Neurosurgery | Percutaneous Vertebroplasty[Including Discography] |
| 2012 | Neurosurgery | Laminectomy, Lumbar Spine |
| 2012 | General surgery | Operation of Hemorrhoids-Hemorrhoidectomy |
| 2012 | General surgery | Appendectomy-Simple |
| 2012 | General surgery | Cholecystectomy |
| 2012 | Urology | Operation For Urinary Incontinence-Transvaginal Approach |
| 2012 | Urology | Transurethral Resection of Prostate |
| 2012 | Urology | Radical Hydrocelectomy |
| 2013 | Orthopedic surgery | Reconstruction of Tendon And Ligament, Simple |
| 2013 | Orthopedic surgery | Menisectomy(Medial or Lateral) |
| 2013 | Orthopedic surgery | Replacement Arthroplasty-Total[Knee] |
| 2013 | Ophthalmology | Intraocular Lens Implantation-Primary |
| 2013 | Ophthalmology | Surgery for Cataract Or Lens-Phacoemulsification |
| 2013 | Ophthalmology | Surgery for Cataract Or Lens-Extracapsular Or Intracapsular Extraction |
| 2013 | Plastic surgery | Trigger Finger |
| 2013 | Plastic surgery | Skin Flap-Local-Others |
| 2013 | Plastic surgery | Escharectomy[9% Under]-Hand, Foot, Finger or Toe |
| 2013 | Dental surgery | Open Reduction of Mandibular Fracture(Symphysis, Body, Angle of Mandible) |
| 2013 | Dental surgery | Operation of Fractured Upper Jaw(Open Reduction, Le Fort Ⅰ) |
| 2013 | Dental surgery | Resection of Tumor of Mandible(Benign Tumor(Including Cyst))-One Side Mandible 1/3 Below |
| 2013 | Obstetrics and gynecology | Cesarean Section Delivery-First Fetus-Initial-Primiparous |
| 2013 | Obstetrics and gynecology | Cesarean Section Delivery-First Fetus-Repeat |
| 2013 | Obstetrics and gynecology | Extirpation of Adnexal Tumor-Benign |
| 2013 | Otorhinolaryngology | Submucosal Resection or Septoplasty(Bone) |
| 2013 | Otorhinolaryngology | Tonsillectomy |
| 2013 | Otorhinolaryngology | Submucosal Inferior Turbinectomy |
| 2013 | Cardiothoracic surgery | Extracorporeal Circulation By Heart-Lung Machine |
| 2013 | Cardiothoracic surgery | Extensive Resection of Varicose Vein-Segmental Stripping of Saphenous Vein,Stab Abulsion of Varices |
| 2013 | Cardiothoracic surgery | Extensive Resection of Varicose Vein-Total Stripping of Saphenous Vein, Stab Abulsion of Varices |
| 2013 | Neurosurgery | Diskectomy(Invasive)-Lumbar Spine |
| 2013 | Neurosurgery | Percutaneous Vertebroplasty[Including Discography] |
| 2013 | Neurosurgery | Laminectomy, Lumbar Spine |
| 2013 | General surgery | Operation of Hemorrhoids-Hemorrhoidectomy |
| 2013 | General surgery | Appendectomy-Simple |
| 2013 | General surgery | Cholecystectomy |
| 2013 | Urology | Operation For Urinary Incontinence-Transvaginal Approach |
| 2013 | Urology | Transurethral Resection of Prostate |
| 2013 | Urology | Radical Hydrocelectomy |
| 2014 | Orthopedic surgery | Reconstruction of Tendon And Ligament, Simple |
| 2014 | Orthopedic surgery | Menisectomy(Medial or Lateral) |
| 2014 | Orthopedic surgery | Replacement Arthroplasty-Total[Knee] |
| 2014 | Ophthalmology | Intraocular Lens Implantation-Primary |
| 2014 | Ophthalmology | Surgery for Cataract Or Lens-Phacoemulsification |
| 2014 | Ophthalmology | Surgery for Cataract Or Lens-Extracapsular Or Intracapsular Extraction |
| 2014 | Plastic surgery | Trigger Finger |
| 2014 | Plastic surgery | Skin Flap-Local-Others |
| 2014 | Plastic surgery | Escharectomy[9% Under]-Hand, Foot, Finger or Toe |
| 2014 | Dental surgery | Open Reduction of Mandibular Fracture(Symphysis, Body, Angle of Mandible) |
| 2014 | Dental surgery | Operation of Fractured Upper Jaw(Open Reduction, Le Fort Ⅰ) |
| 2014 | Dental surgery | Resection of Tumor of Mandible(Benign Tumor(Including Cyst))-One Side Mandible 1/3 Below |
| 2014 | Obstetrics and gynecology | Cesarean Section Delivery-First Fetus-Initial-Primiparous |
| 2014 | Obstetrics and gynecology | Cesarean Section Delivery-First Fetus-Repeat |
| 2014 | Obstetrics and gynecology | Extirpation of Adnexal Tumor-Benign |
| 2014 | Otorhinolaryngology | Submucosal Resection or Septoplasty(Bone) |
| 2014 | Otorhinolaryngology | Submucosal Inferior Turbinectomy |
| 2014 | Otorhinolaryngology | Tonsillectomy |
| 2014 | Cardiothoracic surgery | Extracorporeal Circulation By Heart-Lung Machine |
| 2014 | Cardiothoracic surgery | Extensive Resection of Varicose Vein-Segmental Stripping of Saphenous Vein,Stab Abulsion of Varices |
| 2014 | Cardiothoracic surgery | Wedge Resection of Lung, Single |
| 2014 | Neurosurgery | Diskectomy(Invasive)-Lumbar Spine |
| 2014 | Neurosurgery | Percutaneous Vertebroplasty[Including Discography] |
| 2014 | Neurosurgery | Laminectomy, Lumbar Spine |
| 2014 | General surgery | Operation of Hemorrhoids-Hemorrhoidectomy |
| 2014 | General surgery | Appendectomy-Simple |
| 2014 | General surgery | Cholecystectomy |
| 2014 | Urology | Operation For Urinary Incontinence-Transvaginal Approach |
| 2014 | Urology | Transurethral Resection of Prostate |
| 2014 | Urology | Radical Hydrocelectomy |
| 2015 | Orthopedic surgery | Reconstruction of Tendon And Ligament, Simple |
| 2015 | Orthopedic surgery | Menisectomy(Medial or Lateral) |
| 2015 | Orthopedic surgery | Replacement Arthroplasty-Total[Knee] |
| 2015 | Ophthalmology | Intraocular Lens Implantation-Primary |
| 2015 | Ophthalmology | Surgery for Cataract Or Lens-Phacoemulsification |
| 2015 | Ophthalmology | Surgery for Cataract Or Lens-Extracapsular Or Intracapsular Extraction |
| 2015 | Plastic surgery | Trigger Finger |
| 2015 | Plastic surgery | Skin Flap-Local-Others |
| 2015 | Plastic surgery | Escharectomy[9% Under]-Hand, Foot, Finger or Toe |
| 2015 | Dental surgery | Operation of Fractured Upper Jaw(Open Reduction, Le Fort Ⅰ) |
| 2015 | Dental surgery | Open Reduction of Mandibular Fracture(Symphysis, Body, Angle of Mandible) |
| 2015 | Dental surgery | Resection of Tumor of Mandible(Benign Tumor(Including Cyst))-One Side Mandible 1/3 Below |
| 2015 | Obstetrics and gynecology | Cesarean Section Delivery-First Fetus-Initial-Primiparous |
| 2015 | Obstetrics and gynecology | Cesarean Section Delivery-First Fetus-Repeat |
| 2015 | Obstetrics and gynecology | Extirpation of Adnexal Tumor-Benign |
| 2015 | Otorhinolaryngology | Submucosal Inferior Turbinectomy |
| 2015 | Otorhinolaryngology | Submucosal Resection or Septoplasty(Bone) |
| 2015 | Otorhinolaryngology | Tonsillectomy |
| 2015 | Cardiothoracic surgery | Extracorporeal Circulation By Heart-Lung Machine |
| 2015 | Cardiothoracic surgery | Wedge Resection of Lung, Single |
| 2015 | Cardiothoracic surgery | Mediasternal Lymph Node Dissection |
| 2015 | Neurosurgery | Diskectomy(Invasive)-Lumbar Spine |
| 2015 | Neurosurgery | Percutaneous Vertebroplasty[Including Discography] |
| 2015 | Neurosurgery | Laminectomy, Lumbar Spine |
| 2015 | General surgery | Operation of Hemorrhoids-Hemorrhoidectomy |
| 2015 | General surgery | Appendectomy-Simple |
| 2015 | General surgery | Cholecystectomy |
| 2015 | Urology | Operation For Urinary Incontinence-Transvaginal Approach |
| 2015 | Urology | Transurethral Resection of Prostate |
| 2015 | Urology | Radical Hydrocelectomy |
| 2016 | Orthopedic surgery | Reconstruction of Tendon And Ligament, Simple |
| 2016 | Orthopedic surgery | Menisectomy(Medial or Lateral) |
| 2016 | Orthopedic surgery | Replacement Arthroplasty-Total[Knee] |
| 2016 | Ophthalmology | Intraocular Lens Implantation-Primary |
| 2016 | Ophthalmology | Surgery for Cataract Or Lens-Phacoemulsification |
| 2016 | Ophthalmology | Vitrectomy-Total |
| 2016 | Plastic surgery | Trigger Finger |
| 2016 | Plastic surgery | Skin Flap-Local-Others |
| 2016 | Plastic surgery | Escharectomy[9% Under]-Hand, Foot, Finger or Toe |
| 2016 | Dental surgery | Resection of Tumor of Mandible(Benign Tumor(Including Cyst))-One Side Mandible 1/3 Below |
| 2016 | Dental surgery | Open Reduction of Mandibular Fracture(Symphysis, Body, Angle of Mandible) |
| 2016 | Dental surgery | Operation of Fractured Upper Jaw(Open Reduction, Le Fort Ⅰ) |
| 2016 | Obstetrics and gynecology | Cesarean Section Delivery-First Fetus-Initial-Primiparous |
| 2016 | Obstetrics and gynecology | Cesarean Section Delivery-First Fetus-Repeat |
| 2016 | Obstetrics and gynecology | Extirpation of Adnexal Tumor-Benign |
| 2016 | Otorhinolaryngology | Submucosal Inferior Turbinectomy |
| 2016 | Otorhinolaryngology | Submucosal Resection or Septoplasty(Bone) |
| 2016 | Otorhinolaryngology | Tonsillectomy |
| 2016 | Cardiothoracic surgery | Extracorporeal Circulation By Heart-Lung Machine |
| 2016 | Cardiothoracic surgery | Mediasternal Lymph Node Dissection |
| 2016 | Cardiothoracic surgery | Extensive Resection of Varicose Vein-Total Stripping of Saphenous Vein, Stab Abulsion of Varices |
| 2016 | Neurosurgery | Diskectomy(Invasive)-Lumbar Spine |
| 2016 | Neurosurgery | Percutaneous Vertebroplasty[Including Discography] |
| 2016 | Neurosurgery | Laminectomy, Lumbar Spine |
| 2016 | General surgery | Operation of Hemorrhoids-Hemorrhoidectomy |
| 2016 | General surgery | Cholecystectomy |
| 2016 | General surgery | Appendectomy-Simple |
| 2016 | Urology | Operation For Urinary Incontinence-Transvaginal Approach |
| 2016 | Urology | Transurethral Resection of Prostate |
| 2016 | Urology | Radical Hydrocelectomy |

**(C) Top 3 surgical indications according to the presence of cirrhosis**

| **Year** | **Liver cirrhosis** | **Name of surgery** |
| --- | --- | --- |
| 2012 | No | Intraocular Lens Implantation-Primary |
| 2012 | Yes | Escharectomy[9% Under]-Hand, Foot, Finger or Toe |
| 2012 | No | Surgery for Cataract Or Lens-Phacoemulsification |
| 2012 | Yes | Intraocular Lens Implantation-Primary |
| 2012 | No | Operation of Hemorrhoids-Hemorrhoidectomy |
| 2012 | Yes | Surgery for Cataract Or Lens-Phacoemulsification |
| 2013 | No | Intraocular Lens Implantation-Primary |
| 2013 | Yes | Escharectomy[9% Under]-Hand, Foot, Finger or Toe |
| 2013 | No | Surgery for Cataract Or Lens-Phacoemulsification |
| 2013 | Yes | Intraocular Lens Implantation-Primary |
| 2013 | No | Operation of Hemorrhoids-Hemorrhoidectomy |
| 2013 | Yes | Surgery for Cataract Or Lens-Phacoemulsification |
| 2014 | No | Intraocular Lens Implantation-Primary |
| 2014 | Yes | Escharectomy[9% Under]-Hand, Foot, Finger or Toe |
| 2014 | No | Surgery for Cataract Or Lens-Phacoemulsification |
| 2014 | Yes | Intraocular Lens Implantation-Primary |
| 2014 | No | Operation of Hemorrhoids-Hemorrhoidectomy |
| 2014 | Yes | Surgery for Cataract Or Lens-Phacoemulsification |
| 2015 | No | Intraocular Lens Implantation-Primary |
| 2015 | Yes | Escharectomy[9% Under]-Hand, Foot, Finger or Toe |
| 2015 | No | Surgery for Cataract Or Lens-Phacoemulsification |
| 2015 | Yes | Intraocular Lens Implantation-Primary |
| 2015 | No | Escharectomy[9% Under]-Hand, Foot, Finger or Toe |
| 2015 | Yes | Surgery for Cataract Or Lens-Phacoemulsification |
| 2016 | No | Intraocular Lens Implantation-Primary |
| 2016 | Yes | Escharectomy[9% Under]-Hand, Foot, Finger or Toe |
| 2016 | No | Surgery for Cataract Or Lens-Phacoemulsification |
| 2016 | Yes | Intraocular Lens Implantation-Primary |
| 2016 | No | Escharectomy[9% Under]-Hand, Foot, Finger or Toe |
| 2016 | Yes | Surgery for Cataract Or Lens-Phacoemulsification |

**(D) Top 3 surgical indications according to the presence of cirrhosis and the department of surgery**

| **Year** | **Liver cirrhosis** | **Department of surgery** | **Name of surgery** |
| --- | --- | --- | --- |
| 2012 | Yes | Orthopedic surgery | Menisectomy(Medial or Lateral) |
| 2012 | Yes | Orthopedic surgery | Open Reduction of Fractured Extremity[Femur] |
| 2012 | Yes | Orthopedic surgery | Total Arthroplasty[Hip] |
| 2012 | Yes | Ophthalmology | Intraocular Lens Implantation-Primary |
| 2012 | Yes | Ophthalmology | Surgery for Cataract Or Lens-Phacoemulsification |
| 2012 | Yes | Ophthalmology | Surgery for Cataract Or Lens-Extracapsular Or Intracapsular Extraction |
| 2012 | Yes | Plastic surgery | Escharectomy[9% Under]-Hand, Foot, Finger or Toe |
| 2012 | Yes | Plastic surgery | Skin Flap-Local-Others |
| 2012 | Yes | Plastic surgery | Skin Flap-Local-Face |
| 2012 | Yes | Dental surgery | Alveoloplasty |
| 2012 | Yes | Dental surgery | Open Reduction of Mandibular Fracture(Symphysis, Body, Angle of Mandible) |
| 2012 | Yes | Dental surgery | Operation of Fractured Upper Jaw(Open Reduction, Le Fort Ⅰ) |
| 2012 | Yes | Obstetrics and gynecology | Extirpation of Adnexal Tumor-Benign |
| 2012 | Yes | Obstetrics and gynecology | Dilatation And Curettage |
| 2012 | Yes | Obstetrics and gynecology | Adnexectomy-Bilateral |
| 2012 | Yes | Otorhinolaryngology | Invasive Tracheostomy |
| 2012 | Yes | Otorhinolaryngology | Radical Operation of Malignant Thyroid Tumor |
| 2012 | Yes | Otorhinolaryngology | Submucosal Resection or Septoplasty(Bone) |
| 2012 | Yes | Cardiothoracic surgery | Fistula Formation-Autologus Vein For Hemodialysis |
| 2012 | Yes | Cardiothoracic surgery | Single Lobectomy of Lung |
| 2012 | Yes | Cardiothoracic surgery | Wedge Resection of Lung, Single |
| 2012 | Yes | Neurosurgery | Diskectomy(Invasive)-Lumbar Spine |
| 2012 | Yes | Neurosurgery | Percutaneous Vertebroplasty[Including Discography] |
| 2012 | Yes | Neurosurgery | Laminectomy, Lumbar Spine |
| 2012 | Yes | General surgery | Cholecystectomy |
| 2012 | Yes | General surgery | Hepatectomy-Segmentectomy |
| 2012 | Yes | General surgery | Donor Hepatectomy-Partial Hepatectomy from Living Donor-Lobe(Left,Right) |
| 2012 | Yes | Urology | Radical Hydrocelectomy |
| 2012 | Yes | Urology | Operation For Urinary Incontinence-Transvaginal Approach |
| 2012 | Yes | Urology | Transurethral Resection of Prostate |
| 2013 | Yes | Orthopedic surgery | Menisectomy(Medial or Lateral) |
| 2013 | Yes | Orthopedic surgery | Replacement Arthroplasty-Total[Knee] |
| 2013 | Yes | Orthopedic surgery | Open Reduction of Fractured Extremity[Femur] |
| 2013 | Yes | Ophthalmology | Intraocular Lens Implantation-Primary |
| 2013 | Yes | Ophthalmology | Surgery for Cataract Or Lens-Phacoemulsification |
| 2013 | Yes | Ophthalmology | Surgery for Cataract Or Lens-Extracapsular Or Intracapsular Extraction |
| 2013 | Yes | Plastic surgery | Escharectomy[9% Under]-Hand, Foot, Finger or Toe |
| 2013 | Yes | Plastic surgery | Skin Flap-Local-Others |
| 2013 | Yes | Plastic surgery | Skin Flap-Local-Face |
| 2013 | Yes | Dental surgery | Alveoloplasty |
| 2013 | Yes | Dental surgery | Resection of Malignant Tumor(Hemimandibulectomy) |
| 2013 | Yes | Dental surgery | Partial Mandibulectomy |
| 2013 | Yes | Obstetrics and gynecology | Extirpation of Adnexal Tumor-Benign |
| 2013 | Yes | Obstetrics and gynecology | Dilatation And Curettage |
| 2013 | Yes | Obstetrics and gynecology | Colporrhaphy-Posterior Colporrhaphy |
| 2013 | Yes | Otorhinolaryngology | Invasive Tracheostomy |
| 2013 | Yes | Otorhinolaryngology | Submucosal Resection or Septoplasty(Bone) |
| 2013 | Yes | Otorhinolaryngology | Foreign Body Removal of Ear Canal Or Removal of Impacted Cerumen-Complex |
| 2013 | Yes | Cardiothoracic surgery | Fistula Formation-Autologus Vein For Hemodialysis |
| 2013 | Yes | Cardiothoracic surgery | Extracorporeal Circulation By Heart-Lung Machine |
| 2013 | Yes | Cardiothoracic surgery | Mediasternal Lymph Node Dissection |
| 2013 | Yes | Neurosurgery | Percutaneous Vertebroplasty[Including Discography] |
| 2013 | Yes | Neurosurgery | Diskectomy(Invasive)-Lumbar Spine |
| 2013 | Yes | Neurosurgery | Laminectomy, Lumbar Spine |
| 2013 | Yes | General surgery | Cholecystectomy |
| 2013 | Yes | General surgery | Hepatectomy-Segmentectomy |
| 2013 | Yes | General surgery | Donor Hepatectomy-Partial Hepatectomy from Living Donor-Lobe(Left,Right) |
| 2013 | Yes | Urology | Operation For Urinary Incontinence-Transvaginal Approach |
| 2013 | Yes | Urology | Transurethral Resection of Prostate |
| 2013 | Yes | Urology | Total Prostatoseminal Vesiculectomy |
| 2014 | Yes | Orthopedic surgery | Open Reduction of Fractured Extremity[Femur] |
| 2014 | Yes | Orthopedic surgery | Reconstruction of Tendon And Ligament, Simple |
| 2014 | Yes | Orthopedic surgery | Menisectomy(Medial or Lateral) |
| 2014 | Yes | Ophthalmology | Intraocular Lens Implantation-Primary |
| 2014 | Yes | Ophthalmology | Surgery for Cataract Or Lens-Phacoemulsification |
| 2014 | Yes | Ophthalmology | Vitrectomy-Total |
| 2014 | Yes | Plastic surgery | Escharectomy[9% Under]-Hand, Foot, Finger or Toe |
| 2014 | Yes | Plastic surgery | Skin Flap-Local-Others |
| 2014 | Yes | Plastic surgery | Trigger Finger |
| 2014 | Yes | Dental surgery | Alveoloplasty |
| 2014 | Yes | Dental surgery | Operation of Fractured Upper Jaw(Open Reduction, Le Fort Ⅰ) |
| 2014 | Yes | Dental surgery | Operation of Fractured Lower Jaw(Open Reduction)-Symphysis, Body, Angle of Mandible |
| 2014 | Yes | Obstetrics and gynecology | Extirpation of Adnexal Tumor-Benign |
| 2014 | Yes | Obstetrics and gynecology | Extirpation of Adnexal Tumor(Malignant)-Radical |
| 2014 | Yes | Obstetrics and gynecology | Dilatation And Curettage |
| 2014 | Yes | Otorhinolaryngology | Invasive Tracheostomy |
| 2014 | Yes | Otorhinolaryngology | Foreign Body Removal of Ear Canal Or Removal of Impacted Cerumen-Complex |
| 2014 | Yes | Otorhinolaryngology | Radical Operation of Malignant Thyroid Tumor |
| 2014 | Yes | Cardiothoracic surgery | Fistula Formation-Autologus Vein For Hemodialysis |
| 2014 | Yes | Cardiothoracic surgery | Wedge Resection of Lung, Single |
| 2014 | Yes | Cardiothoracic surgery | Mediasternal Lymph Node Dissection |
| 2014 | Yes | Neurosurgery | Percutaneous Vertebroplasty[Including Discography] |
| 2014 | Yes | Neurosurgery | Diskectomy(Invasive)-Lumbar Spine |
| 2014 | Yes | Neurosurgery | Laminectomy, Lumbar Spine |
| 2014 | Yes | General surgery | Cholecystectomy |
| 2014 | Yes | General surgery | Hepatectomy-Segmentectomy |
| 2014 | Yes | General surgery | Hepatectomy-Lobectomy |
| 2014 | Yes | Urology | Radical Hydrocelectomy |
| 2014 | Yes | Urology | Transurethral Resection of Prostate |
| 2014 | Yes | Urology | Operation For Urinary Incontinence-Transvaginal Approach |
| 2015 | Yes | Orthopedic surgery | Menisectomy(Medial or Lateral) |
| 2015 | Yes | Orthopedic surgery | Reconstruction of Tendon And Ligament, Simple |
| 2015 | Yes | Orthopedic surgery | Replacement Arthroplasty-Total[Knee] |
| 2015 | Yes | Ophthalmology | Intraocular Lens Implantation-Primary |
| 2015 | Yes | Ophthalmology | Surgery for Cataract Or Lens-Phacoemulsification |
| 2015 | Yes | Ophthalmology | Vitrectomy-Total |
| 2015 | Yes | Plastic surgery | Escharectomy[9% Under]-Hand, Foot, Finger or Toe |
| 2015 | Yes | Plastic surgery | Skin Flap-Local-Others |
| 2015 | Yes | Plastic surgery | Trigger Finger |
| 2015 | Yes | Dental surgery | Alveoloplasty |
| 2015 | Yes | Dental surgery | Open Reduction of Fractured Jaw |
| 2015 | Yes | Dental surgery | Open Reduction of Mandibular Fracture(Symphysis, Body, Angle of Mandible) |
| 2015 | Yes | Obstetrics and gynecology | Extirpation of Adnexal Tumor-Benign |
| 2015 | Yes | Obstetrics and gynecology | Dilatation And Curettage |
| 2015 | Yes | Obstetrics and gynecology | Pelvic And Para-Aortic Lymphadenectomy |
| 2015 | Yes | Otorhinolaryngology | Invasive Tracheostomy |
| 2015 | Yes | Otorhinolaryngology | Submucosal Inferior Turbinectomy |
| 2015 | Yes | Otorhinolaryngology | Foreign Body Removal of Ear Canal Or Removal of Impacted Cerumen-Complex |
| 2015 | Yes | Cardiothoracic surgery | Fistula Formation-Autologus Vein For Hemodialysis |
| 2015 | Yes | Cardiothoracic surgery | Mediasternal Lymph Node Dissection |
| 2015 | Yes | Cardiothoracic surgery | Extracorporeal Circulation By Heart-Lung Machine |
| 2015 | Yes | Neurosurgery | Percutaneous Vertebroplasty[Including Discography] |
| 2015 | Yes | Neurosurgery | Diskectomy(Invasive)-Lumbar Spine |
| 2015 | Yes | Neurosurgery | BurrHole or Trephination For Drainage And/Or Evacuation of Cyst,Hematoma or Abscess(Sub or Epidural) |
| 2015 | Yes | General surgery | Cholecystectomy |
| 2015 | Yes | General surgery | Hepatectomy-Lobectomy |
| 2015 | Yes | General surgery | Hepatectomy-Segmentectomy |
| 2015 | Yes | Urology | Transurethral Resection of Prostate |
| 2015 | Yes | Urology | Radical Hydrocelectomy |
| 2015 | Yes | Urology | Operation For Urinary Incontinence-Transvaginal Approach |
| 2016 | Yes | Orthopedic surgery | Replacement Arthroplasty-Total[Knee] |
| 2016 | Yes | Orthopedic surgery | Reconstruction of Tendon And Ligament, Simple |
| 2016 | Yes | Orthopedic surgery | Menisectomy(Medial or Lateral) |
| 2016 | Yes | Ophthalmology | Intraocular Lens Implantation-Primary |
| 2016 | Yes | Ophthalmology | Surgery for Cataract Or Lens-Phacoemulsification |
| 2016 | Yes | Ophthalmology | Vitrectomy-Total |
| 2016 | Yes | Plastic surgery | Escharectomy[9% Under]-Hand, Foot, Finger or Toe |
| 2016 | Yes | Plastic surgery | Skin Flap-Local-Others |
| 2016 | Yes | Plastic surgery | Split Thickness Skin Graft-Others(25㎠∼100㎠) |
| 2016 | Yes | Dental surgery | Alveoloplasty |
| 2016 | Yes | Dental surgery | Resection of Tumor of Mandible(Benign Tumor(Including Cyst)-One Side Mandible 1/3 Below |
| 2016 | Yes | Dental surgery | Operation of Fractured Upper Jaw(Open Reduction, Le Fort Ⅱ) |
| 2016 | Yes | Obstetrics and gynecology | Dilatation And Curettage |
| 2016 | Yes | Obstetrics and gynecology | Extirpation of Adnexal Tumor-Benign |
| 2016 | Yes | Obstetrics and gynecology | Cesarean Section Delivery-First Fetus-Repeat |
| 2016 | Yes | Otorhinolaryngology | Invasive Tracheostomy |
| 2016 | Yes | Otorhinolaryngology | Foreign Body Removal of Ear Canal Or Removal of Impacted Cerumen-Complex |
| 2016 | Yes | Otorhinolaryngology | Tympanoplasty |
| 2016 | Yes | Cardiothoracic surgery | Fistula Formation-Autologus Vein For Hemodialysis |
| 2016 | Yes | Cardiothoracic surgery | Extracorporeal Circulation By Heart-Lung Machine |
| 2016 | Yes | Cardiothoracic surgery | Fistula Formation-Artificial Vein For Hemodialysis |
| 2016 | Yes | Neurosurgery | Diskectomy(Invasive)-Lumbar Spine |
| 2016 | Yes | Neurosurgery | Percutaneous Vertebroplasty[Including Discography] |
| 2016 | Yes | Neurosurgery | BurrHole or Trephination For Drainage And/Or Evacuation of Cyst,Hematoma or Abscess(Sub or Epidural) |
| 2016 | Yes | General surgery | Cholecystectomy |
| 2016 | Yes | General surgery | Hepatectomy-Lobectomy |
| 2016 | Yes | General surgery | Donor Hepatectomy-Partial Hepatectomy from Living Donor-Lobe(Left,Right) |
| 2016 | Yes | Urology | Operation For Urinary Incontinence-Transvaginal Approach |
| 2016 | Yes | Urology | Transurethral Resection of Prostate |
| 2016 | Yes | Urology | Radical Hydrocelectomy |
