# Supplementary material for: Adverse outcomes after surgeries in patients with liver cirrhosis among Korean population: A population-based study
Source: PLoS One. 2021 Jun 14;16(6):e0253165. doi: 10.1371/journal.pone.0253165 (PMC8202950; doi:10.1371/journal.pone.0253165)
Supplement: S5 Table — (DOCX) [file pone.0253165.s005.docx]

**Supplementary Table 3-2. Multivariate logistic regression predicting post-operative complication of all patients**

|  | **Unmatched set (N=1,662,887)** | | | | **Matched set (N=32,194)** | | |  |
| --- | --- | --- | --- | --- | --- | --- | --- | --- |
| **Variables** | **Univariate** | | **Multivariate** | | **Univariate** | | **Multivariate** |  |
|  | **OR (95% CI)** | **p-value** | **OR (95% CI)** | **p-value** | **OR (95% CI)** | **p-value** | **OR (95% CI)** | **p-value** |
| Age (year) | 1(1-1) | <.0001 | 1(1-1) | <.0001 | 1(0.99-1.01) | 0.886 |  |  |
| Sex |  |  |  |  |  |  |  |  |
| Female | 1 (Ref) |  |  |  | 1 (Ref) |  |  |  |
| Male | 1.01(1-1.02) | 0.342 |  |  | 0.88(0.58-1.32) | 0.527 |  |  |
| Medical insurance state |  |  |  |  |  |  |  |  |
| Health insurance | 1 (Ref) |  | 1 (Ref) |  | 1 (Ref) |  |  |  |
| Veterans or medical assistance | 1.06(1.04-1.09) | <.0001 | 1.05(1.02-1.08) | 0.001 | 0.74(0.5-1.08) | 0.122 |  |  |
| Comorbidities (n, %) |  |  |  |  |  |  |  |  |
| Charlson comorbidity index | 1.54(1.54-1.55) | <.0001 | 1.52(1.51-1.53) | <.0001 | 1.66(1.57-1.76) | <.0001 | 1.67(1.57-1.78) | <.0001 |
| Hypertension | 3.78(3.74-3.82) | <.0001 | 1.38(1.36-1.4) | <.0001 | 3.27(2.22-4.83) | <.0001 | 1.57(0.95-2.59) | 0.080 |
| Diabetes | 3.38(3.34-3.42) | <.0001 | 0.54(0.53-0.55) | <.0001 | 1.65(1.17-2.33) | 0.004 | 0.45(0.26-0.78) | 0.004 |
| Malignancy | 2.4(2.35-2.45) | <.0001 | 0.29(0.28-0.3) | <.0001 | 1.18(0.79-1.75) | 0.420 |  |  |
| End stage renal disease | 6.38(5.91-6.88) | <.0001 | 0.46(0.42-0.51) | <.0001 | 0.92(0.42-2.02) | 0.842 |  |  |
| Chronic obstructive pulmonary disease | 7.64(7.44-7.86) | <.0001 | 2.35(2.27-2.43) | <.0001 | 4(2.23-7.19) | <.0001 | 1.68(0.79-3.6) | 0.180 |
| Heart failure | 16.49(16.02-16.96) | <.0001 | 2.65(2.56-2.74) | <.0001 | 5.07(2.86-9) | <.0001 | 1.71(0.84-3.47) | 0.140 |
| Liver cirrhosis | 1.11(1.06-1.16) | <.0001 | 1.08(1.03-1.14) | 0.004 | 1.08(1-1.16) | 0.052 |  |  |
| Hyperlipidemia | 3.4(3.36-3.44) | <.0001 | 1.11(1.09-1.12) | <.0001 | 2.16(1.51-3.09) | <.0001 | 0.9(0.52-1.58) | 0.725 |
| Mental disorder | 2.94(2.91-2.97) | <.0001 | 1.26(1.24-1.27) | <.0001 | 2.04(1.45-2.88) | <.0001 | 1.08(0.67-1.75) | 0.758 |
| Ischemic heart disease | 12.45(12.25-12.65) | <.0001 | 4.81(4.72-4.9) | <.0001 | 3.89(2.52-5.98) | <.0001 | 2.44(1.39-4.29) | 0.002 |
| Parkinson's disease | 4.79(4.53-5.07) | <.0001 | 1.28(1.19-1.37) | <.0001 | 2.44(1.13-5.31) | 0.024 | 3.98(1.24-12.76) | 0.020 |
| Systemic Lupus Erythematosus | 2.32(2.09-2.57) | <.0001 | 0.8(0.7-0.9) | 0.000 | 1(0.25-4) | 1.000 |  |  |
| Level of hospital |  |  |  |  |  |  |  |  |
| Primary hospital (=Clinic) | 1 (Ref) |  | 1 (Ref) |  | 1 (Ref) |  | 1 (Ref) |  |
| Secondary hospital (=Hospital, General hospital) | 1.04(1.02-1.06) | 0.001 | 1.03(1.01-1.05) | 0.011 | 0.65(0.43-0.98) | 0.040 | 0.96(0.51-1.79) | 0.887 |
| Tertiary Hospital | 1.02(0.97-1.08) | 0.435 | 1(0.94-1.06) | 0.912 | 0.36(0.14-0.92) | 0.033 | 0.5(0.15-1.69) | 0.263 |
| Types of anesthesia |  |  |  |  |  |  |  |  |
| Non-General anesthesia | 1 (Ref) |  |  |  | 1 (Ref) |  |  |  |
| General anesthesia | 1.05(1.04-1.06) | <.0001 | 1.04(1.03-1.06) | <.0001 | 0.95(0.66-1.37) | 0.780 |  |  |
| Department of surgery (n, %) |  |  |  |  |  |  |  |  |
| Orthopedic surgery | 1.03(1.02-1.04) | <.0001 | 1.32(1.3-1.35) | <.0001 | 1.11(0.74-1.66) | 0.608 |  |  |
| Ophthalmology | 1.05(1.04-1.07) | <.0001 | 1.36(1.33-1.39) | <.0001 | 0.84(0.52-1.35) | 0.467 |  |  |
| Plastic surgery | 1.06(1.04-1.08) | <.0001 | 1.29(1.26-1.32) | <.0001 | 1.2(0.82-1.76) | 0.337 |  |  |
| Dental surgery | 1.14(1.06-1.23) | 0.001 | 1.35(1.23-1.47) | <.0001 | 0.46(0.18-1.21) | 0.117 |  |  |
| Obstetrics and gynecology | 0.97(0.96-0.99) | 0.000 | 1.3(1.27-1.34) | <.0001 | 0.73(0.29-1.81) | 0.493 |  |  |
| Otorhinolaryngology | 1.02(1-1.04) | 0.023 | 1.33(1.29-1.36) | <.0001 | 1.03(0.63-1.68) | 0.901 |  |  |
| Cardiothoracic surgery | 1.08(1.05-1.11) | <.0001 | 1.29(1.25-1.34) | <.0001 | 0.72(0.44-1.2) | 0.206 |  |  |
| Neurosurgery | 1.06(1.04-1.08) | <.0001 | 1.31(1.28-1.34) | <.0001 | 0.72(0.44-1.17) | 0.181 |  |  |
| General surgery | 1.01(1-1.03) | 0.035 | 1.32(1.29-1.34) | <.0001 | 1.48(1.03-2.12) | 0.032 | 1.44(0.86-2.42) | 0.163 |
| Urology | 1(0.96-1.03) | 0.777 |  |  | 0.5(0.21-1.17) | 0.109 |  |  |

*Matching variable: Age, Sex, Medical insurance state, Hypertension, Diabetes, Malignancy, End stage renal disease, Chronic obstructive pulmonary disease, Heart failure, Hyperlipidemia, Mental disorder, Ischemic heart disease, Parkinson's disease, Systemic Lupus Erythematosus, Level of hospital, Types of anesthesia, Department of surgery
