# Supplementary material for: Adverse outcomes after surgeries in patients with liver cirrhosis among Korean population: A population-based study
Source: PLoS One. 2021 Jun 14;16(6):e0253165. doi: 10.1371/journal.pone.0253165 (PMC8202950; doi:10.1371/journal.pone.0253165)
Supplement: S6 Table — (DOCX) [file pone.0253165.s006.docx]

**Supplementary Table 4-1. Multivariate logistic regression predicting post-operative complication in patients with liver cirrhosis**

| **Patients with liver cirrhosis (N=16,174)** | | | | | |
| --- | --- | --- | --- | --- | --- |
| **Variables** | **Univariate** | | **Multivariate** | | |
|  | **OR (95% CI)** | **p-value** | | **OR (95% CI)** | **p-value** |
| **Age (year)** | 1(0.99-1) | 0.1721 | |  |  |
| **Sex** |  |  | |  |  |
| Female | 1 (Ref) |  | |  |  |
| Male | 0.99(0.89-1.1) | 0.8333 | |  |  |
| **Medical insurance state** |  |  | |  |  |
| Health insurance | 1 (Ref) |  | |  |  |
| Veterans or medical assistance | 0.92(0.79-1.07) | 0.262 | |  |  |
| **Comorbidities (n, %)** | 1.5(1.46-1.54) | <.0001 | | 1.52(1.45-1.6) | <.0001 |
| Charlson comorbidity index |  |  | |  |  |
| Hypertension | 3.43(3.09-3.81) | <.0001 | | 1.26(1.09-1.45) | 0.0023 |
| Diabetes | 2.99(2.65-3.39) | <.0001 | | 0.52(0.43-0.63) | <.0001 |
| Malignancy | 2.14(1.75-2.61) | <.0001 | | 0.26(0.19-0.35) | <.0001 |
| End stage renal disease | 3.93(1.9-8.12) | 0.0002 | | 0.31(0.12-0.79) | 0.0136 |
| Chronic obstructive pulmonary disease | 6.88(5.25-9.03) | <.0001 | | 2.18(1.57-3.05) | <.0001 |
| Heart failure | 16.6(12.4-22.23) | <.0001 | | 2.42(1.7-3.43) | <.0001 |
| Hyperlipidemia | 3.01(2.71-3.34) | <.0001 | | 1.05(0.9-1.22) | 0.5476 |
| Mental disorder | 2.59(2.32-2.9) | <.0001 | | 1.18(1.03-1.36) | 0.0166 |
| Ischemic heart disease | 12.38(10.52-14.57) | <.0001 | | 5.16(4.24-6.28) | <.0001 |
| Parkinson's disease | 5.75(3.39-9.75) | <.0001 | | 1.43(0.74-2.75) | 0.2854 |
| Systemic Lupus Erythematosus | 4.58(1.8-11.64) | 0.0014 | | 2.35(0.82-6.74) | 0.1135 |
| **Level of hospital** |  |  | |  |  |
| Primary hospital | 1 (Ref) |  | |  |  |
| Secondary hospital | 0.92(0.76-1.11) | 0.3553 | |  |  |
| Tertiary Hospital | 1.19(0.75-1.89) | 0.4539 | |  |  |
| **Types of anesthesia** |  |  | |  |  |
| Non-General anesthesia | 1 (Ref) |  | |  |  |
| General anesthesia | 1.09(0.99-1.21) | 0.0702 | |  |  |
| **Severity of liver cirrhosis** |  |  | |  |  |
| Decompensated liver cirrhosis | 2.16(1.85-2.52) | <.0001 | | 1.42(1.16-1.74) | 0.0006 |
| Chronic hepatitis B | 1.38(0.96-1.99) | 0.0867 | |  |  |
| Chronic hepatitis C | 3.63(1.83-7.2) | 0.0002 | | 1.39(0.59-3.31) | 0.4515 |
| Ascites | 1.98(1.47-2.66) | <.0001 | | 1.08(0.74-1.58) | 0.6777 |
| Varices | 1.07(0.88-1.32) | 0.4928 | |  |  |
| Hepatic encephalopathy | 1.68(0.48-5.86) | 0.4124 | |  |  |
| **Department of surgery (n, %)** |  |  | |  |  |
| Orthopedic surgery | 1.21(1.07-1.37) | 0.0021 | | 1.24(1.09-1.42) | 0.0014 |
| Ophthalmology | 1.12(0.97-1.29) | 0.1131 | |  |  |
| Plastic surgery | 1.09(0.98-1.21) | 0.1316 | |  |  |
| Dental surgery | 0.81(0.35-1.88) | 0.6213 | |  |  |
| Obstetrics and gynecology | 0.99(0.68-1.46) | 0.9774 | |  |  |
| Otorhinolaryngology | 1.19(0.98-1.45) | 0.0821 | |  |  |
| Cardiothoracic surgery | 0.95(0.76-1.19) | 0.6568 | |  |  |
| Neurosurgery | 1.08(0.92-1.28) | 0.3618 | |  |  |
| General surgery | 1.03(0.92-1.14) | 0.6397 | |  |  |
| Urology | 0.85(0.56-1.29) | 0.4506 | |  |  |
