# Supplementary material for: Adverse outcomes after surgeries in patients with liver cirrhosis among Korean population: A population-based study
Source: PLoS One. 2021 Jun 14;16(6):e0253165. doi: 10.1371/journal.pone.0253165 (PMC8202950; doi:10.1371/journal.pone.0253165)
Supplement: S7 Table — (DOCX) [file pone.0253165.s007.docx]

**Supplementary Table 4-2. Multivariate logistic regression predicting in-hospital mortality in patients with liver cirrhosis**

| **Patients with liver cirrhosis (N=16,174)** | | | | |
| --- | --- | --- | --- | --- |
| **Variables** | **Univariate** | | **Multivariate** | |
|  | **OR (95% CI)** | **p-value** | **OR (95% CI)** | **p-value** |
| **Age (year)** | 1.01(1.01-1.01) | <.0001 | 1.01(1.01-1.02) | <.0001 |
| **Sex** |  |  |  |  |
| Female | 1 (Ref) |  | 1 (Ref) |  |
| Male | 1.12(1.02-1.22) | 0.0144 | 1.16(1.05-1.27) | 0.0032 |
| **Medical insurance state** |  |  |  |  |
| Health insurance | 1 (Ref) |  |  |  |
| Veterans or medical assistance | 1.09(0.97-1.23) | 0.1419 |  |  |
| **Comorbidities (n, %)** | 1.34(1.31-1.38) | <.0001 | 1.36(1.3-1.41) | <.0001 |
| Charlson comorbidity index |  |  |  |  |
| Hypertension | 2.34(2.13-2.56) | <.0001 | 1.13(1-1.28) | 0.0556 |
| Diabetes | 2.19(1.95-2.44) | <.0001 | 0.63(0.53-0.74) | <.0001 |
| Malignancy | 1.54(1.28-1.85) | <.0001 | 0.34(0.27-0.44) | <.0001 |
| End stage renal disease | 2.55(1.25-5.18) | 0.01 | 0.42(0.18-0.99) | 0.046 |
| Chronic obstructive pulmonary disease | 4.45(3.4-5.82) | <.0001 | 1.88(1.37-2.57) | <.0001 |
| Heart failure | 10.45(7.76-14.07) | <.0001 | 2.41(1.71-3.39) | <.0001 |
| Hyperlipidemia | 2.19(2-2.4) | <.0001 | 1.07(0.94-1.21) | 0.3074 |
| Mental disorder | 1.87(1.69-2.06) | <.0001 | 1.09(0.97-1.22) | 0.1625 |
| Ischemic heart disease | 7.6(6.47-8.93) | <.0001 | 3.94(3.26-4.75) | <.0001 |
| Parkinson's disease | 4.03(2.39-6.78) | <.0001 | 1.33(0.72-2.45) | 0.3585 |
| Systemic Lupus Erythematosus | 2.6(1.02-6.6) | 0.0452 | 1.4(0.5-3.89) | 0.5213 |
| **Level of hospital** |  |  |  |  |
| Primary hospital | 1 (Ref) |  |  |  |
| Secondary hospital | 0.93(0.8-1.09) | 0.3815 |  |  |
| Tertiary Hospital | 1.31(0.9-1.9) | 0.1583 |  |  |
| **Types of anesthesia** |  |  |  |  |
| Non-General anesthesia |  |  |  |  |
| General anesthesia | 0.98(0.9-1.06) | 0.6099 |  |  |
| **Severity of liver cirrhosis** |  |  |  |  |
| Decompensated liver cirrhosis | 1.69(1.47-1.95) | <.0001 | 1.26(1.06-1.5) | 0.009 |
| Chronic hepatitis B | 1.16(0.841.6) | 0.3797 |  |  |
| Chronic hepatitis C | 2.6(1.345.03) | 0.0046 | 1.3(0.6-2.83) | 0.5087 |
| Ascites | 1.52(1.151.99) | 0.0028 | 0.95(0.68-1.33) | 0.7771 |
| Varices | 1.42(1.221.66) | <.0001 | 1.41(1.19-1.66) | <.0001 |
| Hepatic encephalopathy | 1.37(0.454.2) | 0.5842 |  |  |
| **Department of surgery (n, %)** |  |  |  |  |
| Orthopedic surgery | 0.99(0.89-1.09) | 0.7785 |  |  |
| Ophthalmology | 0.76(0.67-0.86) | <.0001 | 0.85(0.73-0.98) | 0.0267 |
| Plastic surgery | 1.28(1.18-1.4) | <.0001 | 1.36(1.23-1.51) | <.0001 |
| Dental surgery | 0.64(0.3-1.33) | 0.2292 |  |  |
| Obstetrics and gynecology | 0.74(0.52-1.04) | 0.0839 |  |  |
| Otorhinolaryngology | 1.67(1.43-1.94) | <.0001 | 1.86(1.58-2.2) | <.0001 |
| Cardiothoracic surgery | 1.32(1.12-1.56) | 0.0009 | 1.56(1.31-1.86) | <.0001 |
| Neurosurgery | 1.57(1.38-1.78) | <.0001 | 1.72(1.49-1.99) | <.0001 |
| General surgery | 0.83(0.76-0.91) | <.0001 | 1.01(0.91-1.12) | 0.8661 |
| Urology | 0.71(0.49-1.01) | 0.0581 |  |  |
